# Supplementary material for: Low‐Intensity Focused Ultrasound Enables Temporal Modulation of Human Midbrain Organoid Differentiation
Source: Adv Sci (Weinh). 2026 Nov 9:e00019. Online ahead of print. doi: 10.1002/advs.202600019 (PMC13748774; doi:10.1002/advs.202600019)
Supplement: Supplementary file 2 [file ADVS-9999-e00019-s002.docx]

Supporting Information

Low-intensity focused ultrasound enables temporal modulation of human midbrain organoid differentiation

Jinseong Jeong^1,^**^†^**, Yehhyun Jo^1,^**^†^**, Youngsun Lee^2,3,^**^†^**, Eunyoung Jang^1^, Yeonji Jeong^4^, Won Do Heo^4^, Jennifer H. Shin^5,6,7^, Mi-Ok Lee^2,3,*^, and Hyunjoo J. Lee^1,8,9,*^

**Supplementary Figures**

**Figure S1. Photos of ultrasound transducer platforms.** (**A**) Integration of 24-well plates and six ultrasound transducer platforms placed inside the incubator. (**B**) Top view of a single ultrasound transducer platform. (**C**) Front view of the ultrasound transducer platform.

**Figure S2. Preliminary data for PRF selection. (A**) Relative mRNA expression of *cFOS* across three different PRFs. 30 Hz, two-sample two-sided unpaired Student’s *t*-test, *t* = -2.703, **p* < 0.05 (*n* = 2 biological replicates). 1 kHz, two-sample two-sided unpaired Student’s *t*-test, *t* = -3.342, **p* < 0.05 (*n* = 3 biological replicates). 3 kHz, two-sample two-sided unpaired Student’s *t*-test, *t* = -3.508, *p* = 0.073 (*n* = 2 biological replicates). (**B**) Relative mRNA expression of *TH* across three different PRFs. 30 Hz, two-sample two-sided unpaired Student’s *t*-test, *t* = 4.227, *p* = 0.052 (*n* = 2 biological replicates). 1 kHz, two-sample two-sided unpaired Student’s *t*-test, *t* = 4.474, **p* < 0.05 (*n* = 3 biological replicates). 3 kHz, two-sample two-sided unpaired Student’s *t*-test, *t* = 2.260, *p* = 0.152 (*n* = 2 biological replicates). Solid bars and error bars indicate the mean value and standard deviation, respectively.

**Figure S3. FEM simulation of the ultrasound beam for optimizing the Well-PZT size. (A)** Single well simulation results of a 96-well plate. **(B)** Single well simulation results of a 24-well plate. **(C)** Single well simulation results of a 6-well plate.

**Figure S4. Robustness test of the platform over 9 operations. (A)** Magnitude of measured electrical impedances. **(B)** Phase of measured electrical impedances.

**Figure S5. Evaluation of ultrasound intensity variations across six platforms.** I_SPPA_ of each platform for generating ultrasound.

**Figure S6. Additional analysis of ultrasound-induced cellular activation.** (**A and B**) Representative immunostaining images of YAP. Scale bar, 20 μm. (**C**) Nuclear translocation ratio of YAP in the US group. MD35, two-sample two-sided unpaired Student’s *t*-test, *t* = –1.338, *p* = 0.239 (*n* = 3 (control), 4 (US) biological replicates, > 3 technical replicates). MD55, two-sample two-sided unpaired Student’s *t*-test, *t* = –3.245, **p* < 0.05 (*n* = 4 biological replicates, > 10 technical replicates). (**D**) Relative mRNA expression of *CTGF* in the US group. MD35, two-sample two-sided unpaired Student’s *t*-test, *t* = –9.235, ****p* < 0.001 (*n* = 2 biological replicates, 3 technical replicates). MD55, Mann-Whitney U test, *U* = 18, *p* = 1 (*n* = 2 biological replicates, 3 technical replicates). Solid bars and error bars indicate the mean value and standard deviation, respectively.

**Figure S7. Characterization of MD1 midbrain organoids.** Representative immunofluorescence images demonstrating the expression of neural stem cell marker (Nestin), stemness marker (SOX2), neuron marker (MAP2), and dopaminergic neuron marker (TH). Scale bar, 50 μm

**Figure S8. TH expression under ultrasound stimulation.** (**A**) Relative mRNA expression of *TH* in the US group. MD35, two-sample two-sided unpaired Student’s *t*-test, *t* = 0.519, *p* = 0.617 (*n* = 2 biological replicates, 3 technical replicates). MD55, two-sample two-sided unpaired Student’s *t*-test, *t* = 2.586, **p* < 0.05 (*n* = 2 biological replicates, 3 technical replicates). (**B**) Relative protein level of TH in the US group. MD35, Mann-Whitney U test, *U* = 12, *p* = 0.343 (*n* = 4 biological replicates, > 5 technical replicates). MD55, two-sample two-sided unpaired Student’s *t*-test, *t* = 0.925, *p* = 0.423 (*n* = 4 biological replicates, > 5 technical replicates). (**C and D**) Representative immunostaining images of TH. Scale bar, 20 μm. Solid bars and error bars indicate the mean value and standard deviation, respectively.

**Figure S9. Analysis of organoid dopamine secretion.** Dopamine concentrations were normalized to the control group. On MD35, one sample Student’s *t*-test, *t* = -2.295, *p* = 0.149 (*n* = 3 biological replicates, 2 technical replicates). On MD55, one sample Student’s *t*-test, *t* = -3.739, *p* = 0.065 (*n* = 3 biological replicates, 2 technical replicates). Error bars indicate standard deviation.

**Figure S10. Effects of ultrasound stimulation on neuronal differentiation.** (**A**) Relative mRNA expression of *MAP2* in the US group. MD35, two-sample two-sided unpaired Student’s *t*-test, *t* = 0.519, *p* = 0.617 (*n* = 2 biological replicates, 3 technical replicates). MD55, two-sample two-sided unpaired Student’s *t*-test, *t* = -3.988, ***p* < 0.01 (*n* = 2 biological replicates, 3 technical replicates). (**B**) Relative protein level of MAP2 in the US group. MD35, two-sample two-sided unpaired Student’s *t*-test, *t* = 1.452, *p* = 0.240 (*n* = 3 (control), 4 (US) biological replicates, > 5 technical replicates). MD55, two-sample two-sided unpaired Student’s *t*-test, *t* = -1.320, *p* = 0.236 (*n* = 4 biological replicates, > 5 technical replicates). (**C and D**) Representative immunostaining images of MAP2. Scale bar, 50 μm. (E and F) Representative immunostaining images of vGLUT1 and GABA. Scale bar, 50 μm. (G) Relative protein level of vGLUT1 in the US group. MD35, two-sample two-sided unpaired Student’s *t*-test, *t* = -1.622, *p* = 0.156 (*n* = 4 biological replicates, > 5 technical replicates). MD55, two-sample two-sided unpaired Student’s *t*-test, *t* = -0.783, *p* = 0.463 (*n* = 4 biological replicates, > 10 technical replicates). (H) Relative protein level of GABA in the US group. MD35, two-sample two-sided unpaired Student’s *t*-test, *t* = -0.948, *p* = 0.380 (*n* = 4 biological replicates, > 5 technical replicates). MD55, two-sample two-sided unpaired Student’s *t*-test, *t* = -1.294, *p* = 0.243 (*n* = 4 biological replicates, > 10 technical replicates). Solid bars and error bars indicate the mean value and standard deviation, respectively.

**Figure S11. Effects of ultrasound stimulation on the growth of midbrain organoids.** (**A**) Widefield images of midbrain organoids. Scale bar, 250 μm (MD2), 500 μm (MD40), and 500 μm (MD55). (**B**) Size of the midbrain organoids (n = 5 (MD40 Control, MD55), 6

(MD2, MD40 US) biological replicates). Solid bars and error bars indicate the mean value and standard deviation, respectively.

**Figure S12. Whole organoid images**. (**A**) Representative immunostaining images of cC3 and p53. Scale bar, 200 μm. (**B and C**) Representative immunostaining images of GFAP and MAP2. Scale bar, 200 μm. (**D and E**) Representative immunostaining images of LMX1A and FOXA2. Scale bar, 200 μm. (**F and G**) Representative immunostaining images of TH. Scale bar, 200 μm.

**Figure S13. Total transcriptome analysis of PSCs.** (**A**) Heatmap plot for DEGs. (**B**) GO analysis of the PSCs and mBOs. (**C**) KEGG pathway analysis of the mBOs and PSCs.

**Figure S14. Measurement of transient temperature changes at the surface of the ultrasound transducer platform.** (**A**) Temperature measurement setup in the incubator. (**B**) Temperature measurement results over 20 minutes.

**Figure S15. Effects of ultrasound stimulation on astrocytic differentiation**. (**A**) Relative mRNA expression of *S100β* in the US group. MD35, two-sample two-sided unpaired Student’s *t*-test, *t* = -5.420, ****p* < 0.001 (*n* = 2 biological replicates, 3 technical replicates). MD55, Mann-Whitney U test, *U* = 26, *p* = 0.240 (*n* = 2 biological replicates, 3 technical replicates). (**B**) Relative protein level of GFAP in the US group. MD35, Mann-Whitney U test, *U* = 9, *p* = 0.4 (*n* = 3 (control), 4 (US) biological replicates, > 5 technical replicates). MD55, Mann-Whitney U test, *U* = 4, *p* = 0.343 (*n* = 4 biological replicates, > 5 technical replicates). (**C and D**) Representative immunostaining images of GFAP. Scale bar, 50 μm. Solid bars and error bars indicate the mean value and standard deviation, respectively.

**Figure S16. Reversibility of ultrasound stimulation**. (**A**) Relative mRNA expression of *cFOS* in the US group. MD100, Mann-Whitney U test, *U* = 17, *p* = 0.937 (*n* = 2 biological replicates, 3 technical replicates). (**B**) Relative mRNA expression of *TH* in the US group. MD100, two-sample two-sided unpaired Student’s *t*-test, *t* = -0.304, *p* = 0.768 (*n* = 2 biological replicates, 3 technical replicates). (**C**) Relative mRNA expression of *LMX1A* in the US group. MD100, Mann-Whitney U test, *U* = 0, ***p* < 0.01 (*n* = 2 biological replicates, 3 technical replicates). (**D**) Relative mRNA expression of *CTGF* in the US group. MD100, two-sample two-sided unpaired Student’s *t*-test, *t* = -5.819, ***p* < 0.01 (*n* = 2 biological replicates, 3 technical replicates). (**E**) Relative mRNA expression of *MAP2* in the US group. MD100, Mann-Whitney U test, *U* = 0, ***p* < 0.01 (*n* = 2 biological replicates, 3 technical replicates). (**F**) Relative mRNA expression of *S100β* in the US group. MD100, two-sample two-sided unpaired Student’s *t*-test, *t* = -8.221, ****p* < 0.001 (*n* = 2 biological replicates, 3 technical replicates). Solid bars and error bars indicate the mean value and standard deviation, respectively.

**Supplementary data**

**Data S1. (separate file)**

Raw data file for ICC analysis of MD35 organoids

**Data S2. (separate file)**

Raw data file for ICC analysis of MD55 organoids

**Data S3. (separate file)**

Raw data file for qPCR

**Data S4. (separate file)**

Raw data file for ELISA

**Data S5. (separate file)**

Raw data file for size measurements

**Data S6. (separate file)**

Raw data file for calcium imaging

**Data S7. (separate file)**

Raw data file for TUNEL assay

**Data S8. (separate file)**

Raw data file for bulk RNA sequencing
